# (2,2′-Bipyridine)bis­(3-carboxy­pyrazine-2-carboxyl­ato)copper(II) dihydrate

**DOI:** 10.1107/S1600536808022885

**Published:** 2008-07-26

**Authors:** Hossein Aghabozorg, Mahdieh Parvizi, Elahe Sadrkhanlou

**Affiliations:** a Department of Chemistry, Tarbiat Moallem University, 49 Mofateh Avenue, Tehran, Iran; b Department of Chemistry, Islamic Azad University, North Tehran Branch, Tehran, Iran; c Department of Biology, Faculty of Science, Shahed University, Opposite Imam Khomeini’s Shrine, Tehran-Qom Highway, Tehran, Iran

## Abstract

The title six-coordinated distorted octa­hedral complex, [Cu(C_6_H_3_N_2_O_4_)_2_(C_10_H_8_N_2_)]·2H_2_O, consists of two 3-carboxy­pyrazine-2-carboxyl­ate anions and one 2,2′-bipyridine ligand. There is a twofold rotation axis positioned at the Cu^II^ center. The N atoms of the pyrazine ring occupy the axial positions and two proton-transferred O atoms of tbe acid together with the two N atoms of the 2,2′-bipyridine ligand complete the equatorial plane. The inter­actions existing in the crystal structure are inter­molecular O—H⋯O hydrogen bonds, and C—H⋯O and C—O⋯π inter­actions (O⋯π =3.145 Å, C—O⋯π = 149.75°).

## Related literature

There are several compounds made from pyrazine-2,3-dicarboxylic acid, but most of them are in a polymeric form; see, for example: Tombul *et al.* (2007 ▶, 2008 ▶). For related literature, see: Egli & Sarkhel (2007 ▶).
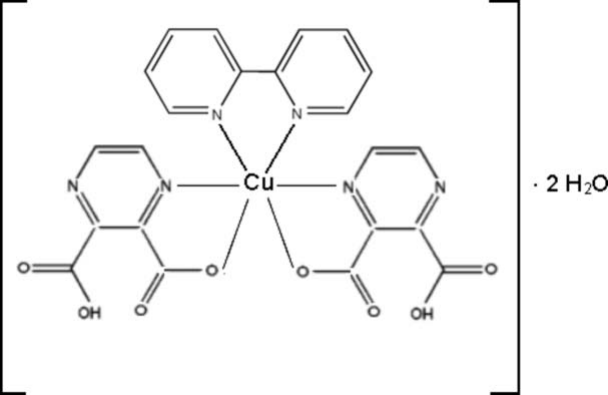


## Experimental

### 

#### Crystal data

[Cu(C_6_H_3_N_2_O_4_)_2_(C_10_H_8_N_2_)]·2H_2_O
*M* *_r_* = 589.96
Monoclinic, 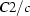

*a* = 18.3080 (8) Å
*b* = 9.2168 (4) Å
*c* = 16.3235 (7) Å
β = 122.480 (5)°
*V* = 2323.59 (18) Å^3^
*Z* = 4
Mo *K*α radiation
μ = 1.01 mm^−1^
*T* = 100 (2) K
0.20 × 0.20 × 0.20 mm

#### Data collection

Bruker SMART APEXII diffractometer
Absorption correction: multi-scan (*SADABS*; Sheldrick, 1996 ▶) *T*_min_ = 0.823, *T*_max_ = 0.823
14982 measured reflections
3500 independent reflections
3289 reflections with *I* > 2σ(*I*)
*R*_int_ = 0.017

#### Refinement

*R*[*F* ^2^ > 2σ(*F* ^2^)] = 0.023
*wR*(*F* ^2^) = 0.063
*S* = 1.04
3500 reflections
177 parameters
H-atom parameters constrained
Δρ_max_ = 0.49 e Å^−3^
Δρ_min_ = −0.31 e Å^−3^

### 

Data collection: *APEX2* (Bruker, 2007 ▶); cell refinement: *SAINT* (Bruker, 2007 ▶); data reduction: *SAINT*; program(s) used to solve structure: *SHELXTL* (Sheldrick, 2008 ▶); program(s) used to refine structure: *SHELXTL*; molecular graphics: *SHELXTL*; software used to prepare material for publication: *SHELXTL*.

## Supplementary Material

Crystal structure: contains datablocks I, global. DOI: 10.1107/S1600536808022885/om2248sup1.cif

Structure factors: contains datablocks I. DOI: 10.1107/S1600536808022885/om2248Isup2.hkl

Additional supplementary materials:  crystallographic information; 3D view; checkCIF report

## Figures and Tables

**Table 1:** Hydrogen-bond geometry (Å, °)

*D*—H⋯*A*	*D*—H	H⋯*A*	*D*⋯*A*	*D*—H⋯*A*
O3—H3O⋯O5^i^	0.88	1.66	2.5390 (12)	170
O5—H5*A*⋯O1	0.84	1.89	2.7218 (13)	173
O5—H5*B*⋯O4^ii^	0.86	1.84	2.6989 (13)	177
C7—H7*A*⋯O2^ii^	0.95	2.57	3.1144 (14)	117
C8—H8*A*⋯O2^ii^	0.95	2.45	3.0433 (13)	121
C9—H9*A*⋯O5^iii^	0.95	2.55	3.2168 (13)	127

Symmetry codes: (i) 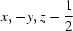; (ii) 
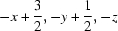
; (iii) 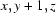.

**Fig. 1.**
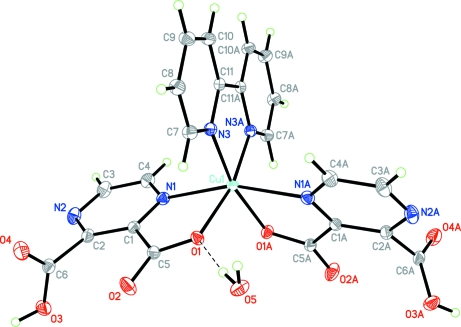
The title compound, with displacement ellipsoids drawn at the 50% probability level.

**Fig. 2.**
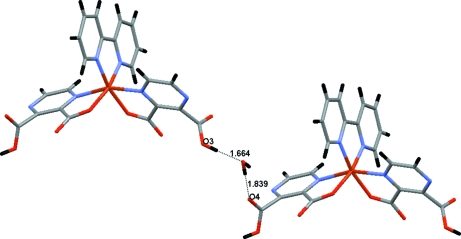
Graph set descriptor of chains made by hydrogen bonding.

**Fig. 3.**
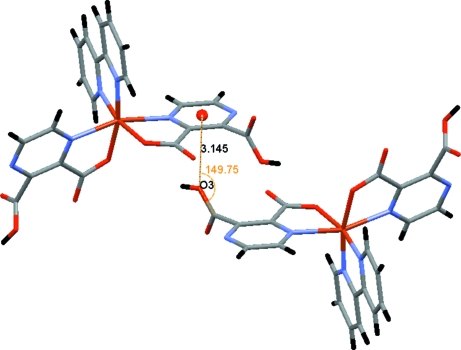
C–O···π interaction between complexes.

**Fig. 4.**
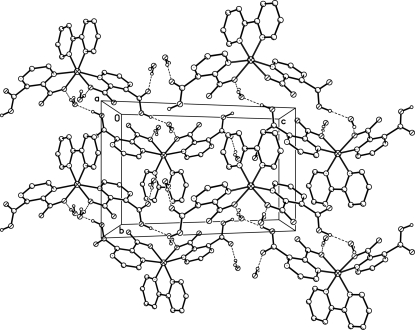
Crystal packing of [Cu(pzdcH)2(2,2'- bpy)]. 2H2O, along c axis.

## supplementary crystallographic information

### Comment

The structure consists of [Cu(2,3-pzdcH)_2_(2,2'- bpy)]. 2H_2_O, where pzdcH_2_ and 2,2'-bpy are pyrazine-2,3-dicarboxylic acid and 2,2'-bipyridine, respectively. The presence of bidentate mono anionic (pzdcH)^-^ and neutral 2,2'-bipyridine ligands results in a neutral complex. The asymmetric unit is given in Fig. 1. The obtained hexacoordinated geometry is distorted octahedral. The bond lengths and angles around the Cu^II^ center are all in accordance with the geometrical steric effects.

There is a 2-fold rotation axis positioned at Cu^II^ center, transforming one half of the compound to the other.

The main interaction responsible for stabilizing such a framework is O–H···O hydrogen bonds. The water molecule participates in two hydrogen bonds relating two neighboring complexes. There is also a weaker C–H···O which joins the third complex to the series.

The O3–H3O···O5(*x*, -*y*, *z* - 1/2, 1.664 Å) and O5–H5B···O4 (-*x* + 3/2, -*y* + 1/2, -*z*, 1.839 Å) form hydrogen-bonded chains described by C^2^_2_(14) graph-set descriptor (Fig. 2). Expansion of these chains results in layer. Furthermore, the C–H···O interactions between the complexes themselves help in the stabilization of the layers. In the third dimension, there is a similar layer at about a 4.1 Å distance. These layers are connected to each other *via *a fascinating C–O···π interaction by C6–O3 and a pyrazine ring (Fig. 3). All factors including O···π distance (3.145 Å), C–O···π angle (α = 149.75°) and dihedral angle between the planes defined by *X*_2_C–O and the aromatic system (ω = 79.18°), are in the mentioned range as in the reference [Egli & Sarkhel, 2007].

Figure 4 represents the packing diagram of this crystal lattice.

### Experimental

To a 10 ml of a stirring aqueous solution of pyrazine-2,3-dicarboxylic acid (0.084 g, 0.5 mmol) and 2,2'- bipyridine (0.078 g, 0.5 mmol), was added a 0.5 molar equivalent of CuSO_4_. 5 H_2_O (0.062 g, 0.25 mmol) at room temperature. A neutral copper(II) complex, [Cu(pzdcH)_2_(2,2'- bpy)]. 2H_2_O, was isolated as very light blue crystals. Slow evaporation of the solvent result in product complex in a week.

### Crystal data

**Table:**

[Cu(C_6_H_3_N_2_O_4_)_2_(C_10_H_8_N_2_)]·2H_2_O	*F*_000_ = 1204
*M**_r_* = 589.96	*D*_x_ = 1.686 Mg m^−^^3^
Monoclinic, *C*2/*c*	Mo *K*α radiation λ = 0.71073 Å
Hall symbol: -C 2yc	Cell parameters from 9557 reflections
*a* = 18.3080 (8) Å	θ = 2.6–30.5º
*b* = 9.2168 (4) Å	µ = 1.01 mm^−^^1^
*c* = 16.3235 (7) Å	*T* = 100 (2) K
β = 122.480 (5)º	Prism, colourless
*V* = 2323.59 (18) Å^3^	0.20 × 0.20 × 0.20 mm
*Z* = 4	

### Data collection

**Table:**

Bruker SMART APEXII diffractometer	3500 independent reflections
Radiation source: fine-focus sealed tube	3289 reflections with *I* > 2σ(*I*)
Monochromator: graphite	*R*_int_ = 0.017
*T* = 100(2) K	θ_max_ = 30.5º
φ and ω scans	θ_min_ = 2.6º
Absorption correction: multi-scan(SADABS; Sheldrick, 1996)	*h* = −26→26
*T*_min_ = 0.823, *T*_max_ = 0.823	*k* = −13→13
14982 measured reflections	*l* = −23→23

### Refinement

**Table:**

Refinement on *F*^2^	Secondary atom site location: difference Fourier map
Least-squares matrix: full	Hydrogen site location: mixed
*R*[*F*^2^ > 2σ(*F*^2^)] = 0.023	H-atom parameters constrained
*wR*(*F*^2^) = 0.063	*w* = 1/[σ^2^(*F*_o_^2^) + (0.04*P*)^2^ + 1.2*P*] where *P* = (*F*_o_^2^ + 2*F*_c_^2^)/3
*S* = 1.04	(Δ/σ)_max_ = 0.001
3500 reflections	Δρ_max_ = 0.49 e Å^−^^3^
177 parameters	Δρ_min_ = −0.31 e Å^−^^3^
Primary atom site location: structure-invariant direct methods	Extinction correction: none

### Special details

**Table:**

Geometry. All e.s.d.'s (except the e.s.d. in the dihedral angle between two l.s. planes) are estimated using the full covariance matrix. The cell e.s.d.'s are taken into account individually in the estimation of e.s.d.'s in distances, angles and torsion angles; correlations between e.s.d.'s in cell parameters are only used when they are defined by crystal symmetry. An approximate (isotropic) treatment of cell e.s.d.'s is used for estimating e.s.d.'s involving l.s. planes.
Refinement. Refinement of *F*^2^ against ALL reflections. The weighted *R*-factor *wR* and goodness of fit *S* are based on *F*^2^, conventional *R*-factors *R* are based on *F*, with *F* set to zero for negative *F*^2^. The threshold expression of *F*^2^ > σ(*F*^2^) is used only for calculating *R*-factors(gt) *etc*. and is not relevant to the choice of reflections for refinement. *R*-factors based on *F*^2^ are statistically about twice as large as those based on *F*, and *R*- factors based on ALL data will be even larger.

### Fractional atomic coordinates and isotropic or equivalent isotropic displacement parameters (Å^2^)

**Table:**

	*x*	*y*	*z*	*U*_iso_*/*U*_eq_	
Cu1	1.0000	0.367362 (16)	0.2500	0.01104 (5)	
O1	0.91326 (4)	0.21893 (8)	0.16501 (5)	0.01386 (13)	
O2	0.85176 (5)	0.08782 (9)	0.02861 (6)	0.02271 (16)	
O3	0.90660 (5)	0.00760 (8)	−0.12364 (6)	0.02014 (15)	
H3O	0.8658	−0.0282	−0.1804	0.024*	
O4	0.81385 (5)	0.19258 (9)	−0.16881 (6)	0.02211 (16)	
N1	1.02854 (5)	0.32913 (9)	0.12702 (6)	0.01404 (15)	
N2	1.01283 (6)	0.27792 (10)	−0.05007 (6)	0.01888 (17)	
N3	0.91587 (5)	0.53313 (8)	0.19274 (6)	0.01230 (14)	
C1	0.96492 (6)	0.24626 (10)	0.05929 (7)	0.01255 (15)	
C2	0.95681 (6)	0.22146 (10)	−0.02994 (7)	0.01416 (16)	
C3	1.07722 (7)	0.35806 (12)	0.01940 (8)	0.01958 (19)	
H3A	1.1187	0.3983	0.0079	0.023*	
C4	1.08554 (7)	0.38463 (11)	0.10811 (8)	0.01748 (18)	
H4A	1.1321	0.4426	0.1556	0.021*	
C5	0.90413 (6)	0.17769 (10)	0.08456 (7)	0.01334 (16)	
C6	0.88379 (7)	0.13731 (10)	−0.11323 (7)	0.01512 (17)	
C7	0.82914 (6)	0.52122 (10)	0.13793 (7)	0.01459 (16)	
H7A	0.8039	0.4274	0.1197	0.018*	
C8	0.77532 (6)	0.64164 (10)	0.10722 (7)	0.01620 (18)	
H8A	0.7142	0.6307	0.0692	0.019*	
C9	0.81274 (6)	0.77869 (11)	0.13324 (7)	0.01659 (17)	
H9A	0.7773	0.8630	0.1126	0.020*	
C10	0.90229 (6)	0.79146 (10)	0.18959 (7)	0.01464 (17)	
H10A	0.9289	0.8843	0.2076	0.018*	
C11	0.95228 (5)	0.66600 (10)	0.21923 (6)	0.01133 (15)	
O5	0.80282 (5)	0.09628 (8)	0.20844 (6)	0.01843 (14)	
H5A	0.8335	0.1329	0.1897	0.022*	
H5B	0.7644	0.1614	0.1957	0.022*	

### Atomic displacement parameters (Å^2^)

**Table:**

	*U*^11^	*U*^22^	*U*^33^	*U*^12^	*U*^13^	*U*^23^
Cu1	0.01075 (8)	0.00986 (8)	0.01153 (8)	0.000	0.00533 (6)	0.000
O1	0.0162 (3)	0.0146 (3)	0.0136 (3)	−0.0032 (2)	0.0099 (3)	−0.0027 (2)
O2	0.0255 (4)	0.0255 (4)	0.0199 (4)	−0.0116 (3)	0.0140 (3)	−0.0098 (3)
O3	0.0209 (3)	0.0171 (3)	0.0184 (3)	0.0053 (3)	0.0079 (3)	−0.0022 (3)
O4	0.0193 (3)	0.0250 (4)	0.0179 (3)	0.0082 (3)	0.0072 (3)	−0.0017 (3)
N1	0.0152 (3)	0.0137 (3)	0.0149 (4)	0.0000 (3)	0.0092 (3)	0.0009 (3)
N2	0.0181 (4)	0.0255 (4)	0.0168 (4)	0.0038 (3)	0.0118 (3)	0.0035 (3)
N3	0.0119 (3)	0.0124 (3)	0.0118 (3)	0.0001 (3)	0.0058 (3)	0.0001 (3)
C1	0.0141 (4)	0.0121 (4)	0.0132 (4)	0.0018 (3)	0.0085 (3)	0.0013 (3)
C2	0.0149 (4)	0.0154 (4)	0.0132 (4)	0.0040 (3)	0.0082 (3)	0.0019 (3)
C3	0.0171 (4)	0.0257 (5)	0.0204 (5)	0.0016 (3)	0.0131 (4)	0.0045 (4)
C4	0.0156 (4)	0.0196 (4)	0.0184 (4)	−0.0010 (3)	0.0099 (4)	0.0018 (3)
C5	0.0146 (4)	0.0134 (4)	0.0133 (4)	−0.0003 (3)	0.0083 (3)	−0.0002 (3)
C6	0.0178 (4)	0.0169 (4)	0.0133 (4)	0.0035 (3)	0.0101 (4)	0.0005 (3)
C7	0.0122 (4)	0.0142 (4)	0.0154 (4)	−0.0007 (3)	0.0060 (3)	−0.0004 (3)
C8	0.0120 (4)	0.0171 (4)	0.0162 (4)	0.0008 (3)	0.0054 (3)	−0.0010 (3)
C9	0.0138 (4)	0.0149 (4)	0.0171 (4)	0.0027 (3)	0.0057 (3)	−0.0008 (3)
C10	0.0144 (4)	0.0122 (4)	0.0153 (4)	0.0006 (3)	0.0067 (3)	−0.0010 (3)
C11	0.0114 (4)	0.0126 (4)	0.0100 (4)	0.0000 (3)	0.0057 (3)	0.0003 (3)
O5	0.0213 (3)	0.0156 (3)	0.0244 (4)	0.0041 (3)	0.0163 (3)	0.0056 (3)

### Geometric parameters (Å, °)

**Table:**

Cu1—O1	1.9880 (7)	C2—C6	1.5117 (14)
Cu1—N3	2.0085 (8)	C3—C4	1.3940 (15)
Cu1—N1	2.3565 (8)	C3—H3A	0.9500
O1—C5	1.2890 (11)	C4—H4A	0.9500
O2—C5	1.2243 (12)	C7—C8	1.3869 (13)
O3—C6	1.3072 (12)	C7—H7A	0.9500
O3—H3O	0.8844	C8—C9	1.3903 (13)
O4—C6	1.2142 (12)	C8—H8A	0.9500
N1—C1	1.3340 (12)	C9—C10	1.3886 (13)
N1—C4	1.3386 (12)	C9—H9A	0.9500
N2—C3	1.3362 (15)	C10—C11	1.3904 (13)
N2—C2	1.3388 (12)	C10—H10A	0.9500
N3—C7	1.3446 (11)	C11—C11^i^	1.4754 (17)
N3—C11	1.3496 (12)	O5—H5A	0.8419
C1—C2	1.4012 (13)	O5—H5B	0.8612
C1—C5	1.5169 (12)		
			
O1—Cu1—O1^i^	93.03 (4)	C4—C3—H3A	118.9
O1—Cu1—N3^i^	166.86 (3)	N1—C4—C3	120.57 (10)
O1—Cu1—N3	94.19 (3)	N1—C4—H4A	119.7
O1^i^—Cu1—N3	166.86 (3)	C3—C4—H4A	119.7
N3^i^—Cu1—N3	80.95 (4)	O2—C5—O1	125.49 (9)
O1—Cu1—N1^i^	91.84 (3)	O2—C5—C1	118.30 (8)
N3—Cu1—N1^i^	92.57 (3)	O1—C5—C1	116.20 (8)
O1—Cu1—N1	76.24 (3)	O4—C6—O3	124.67 (10)
N3—Cu1—N1	100.53 (3)	O4—C6—C2	121.68 (9)
N1^i^—Cu1—N1	162.80 (4)	O3—C6—C2	113.34 (8)
C5—O1—Cu1	122.13 (6)	N3—C7—C8	122.08 (9)
C6—O3—H3O	109.2	N3—C7—H7A	119.0
C1—N1—C4	118.00 (9)	C8—C7—H7A	119.0
C1—N1—Cu1	107.29 (6)	C7—C8—C9	118.61 (9)
C4—N1—Cu1	134.12 (7)	C7—C8—H8A	120.7
C3—N2—C2	116.72 (9)	C9—C8—H8A	120.7
C7—N3—C11	119.40 (8)	C10—C9—C8	119.48 (9)
C7—N3—Cu1	125.73 (6)	C10—C9—H9A	120.3
C11—N3—Cu1	114.69 (6)	C8—C9—H9A	120.3
N1—C1—C2	120.85 (8)	C9—C10—C11	118.84 (9)
N1—C1—C5	117.05 (8)	C9—C10—H10A	120.6
C2—C1—C5	122.07 (8)	C11—C10—H10A	120.6
N2—C2—C1	121.62 (9)	N3—C11—C10	121.57 (8)
N2—C2—C6	113.75 (8)	N3—C11—C11^i^	114.75 (5)
C1—C2—C6	124.57 (8)	C10—C11—C11^i^	123.67 (5)
N2—C3—C4	122.22 (9)	H5A—O5—H5B	104.5
N2—C3—H3A	118.9		
			
O1^i^—Cu1—O1—C5	−96.65 (7)	C3—N2—C2—C6	177.66 (9)
N3^i^—Cu1—O1—C5	26.65 (17)	N1—C1—C2—N2	0.90 (14)
N3—Cu1—O1—C5	94.33 (7)	C5—C1—C2—N2	−177.34 (9)
N1^i^—Cu1—O1—C5	−172.96 (7)	N1—C1—C2—C6	−175.91 (9)
N1—Cu1—O1—C5	−5.49 (7)	C5—C1—C2—C6	5.84 (14)
O1—Cu1—N1—C1	8.79 (6)	C2—N2—C3—C4	−1.13 (15)
O1^i^—Cu1—N1—C1	101.46 (6)	C1—N1—C4—C3	1.09 (14)
N3^i^—Cu1—N1—C1	−164.26 (6)	Cu1—N1—C4—C3	−168.92 (7)
N3—Cu1—N1—C1	−82.99 (6)	N2—C3—C4—N1	0.34 (16)
N1^i^—Cu1—N1—C1	55.98 (14)	Cu1—O1—C5—O2	−179.47 (8)
O1—Cu1—N1—C4	179.57 (10)	Cu1—O1—C5—C1	1.41 (11)
O1^i^—Cu1—N1—C4	−87.76 (10)	N1—C1—C5—O2	−171.48 (9)
N3^i^—Cu1—N1—C4	6.52 (10)	C2—C1—C5—O2	6.83 (14)
N3—Cu1—N1—C4	87.79 (10)	N1—C1—C5—O1	7.71 (12)
N1^i^—Cu1—N1—C4	−133.24 (9)	C2—C1—C5—O1	−173.98 (9)
O1—Cu1—N3—C7	15.90 (8)	N2—C2—C6—O4	−96.00 (11)
O1^i^—Cu1—N3—C7	−107.29 (14)	C1—C2—C6—O4	81.03 (13)
N3^i^—Cu1—N3—C7	−176.39 (10)	N2—C2—C6—O3	77.89 (11)
N1^i^—Cu1—N3—C7	−76.14 (8)	C1—C2—C6—O3	−105.07 (11)
N1—Cu1—N3—C7	92.66 (8)	C11—N3—C7—C8	0.14 (14)
O1—Cu1—N3—C11	−169.01 (6)	Cu1—N3—C7—C8	175.02 (7)
O1^i^—Cu1—N3—C11	67.81 (15)	N3—C7—C8—C9	0.79 (15)
N3^i^—Cu1—N3—C11	−1.30 (5)	C7—C8—C9—C10	−0.57 (15)
N1^i^—Cu1—N3—C11	98.96 (6)	C8—C9—C10—C11	−0.53 (15)
N1—Cu1—N3—C11	−92.25 (7)	C7—N3—C11—C10	−1.31 (13)
C4—N1—C1—C2	−1.68 (14)	Cu1—N3—C11—C10	−176.74 (7)
Cu1—N1—C1—C2	170.83 (7)	C7—N3—C11—C11^i^	178.90 (9)
C4—N1—C1—C5	176.65 (8)	Cu1—N3—C11—C11^i^	3.47 (12)
Cu1—N1—C1—C5	−10.84 (9)	C9—C10—C11—N3	1.50 (14)
C3—N2—C2—C1	0.53 (14)	C9—C10—C11—C11^i^	−178.73 (10)

Symmetry codes: (i) −*x*+2, *y*, −*z*+1/2.

### Hydrogen-bond geometry (Å, °)

**Table:**

*D*—H···*A*	*D*—H	H···*A*	*D*···*A*	*D*—H···*A*
O3—H3O···O5^ii^	0.88	1.66	2.5390 (12)	170
O5—H5A···O1	0.84	1.89	2.7218 (13)	173
O5—H5B···O4^iii^	0.86	1.84	2.6989 (13)	177
C7—H7A···O2^iii^	0.95	2.57	3.1144 (14)	117
C8—H8A···O2^iii^	0.95	2.45	3.0433 (13)	121
C9—H9A···O5^iv^	0.95	2.55	3.2168 (13)	127

Symmetry codes: (ii) *x*, −*y*, *z*−1/2; (iii) −*x*+3/2, −*y*+1/2, −*z*; (iv) *x*, *y*+1, *z*.
